# Is the sex ratio of Japanese quail offspring equal?

**DOI:** 10.1007/s11250-024-04224-3

**Published:** 2024-11-16

**Authors:** Ahmed M. Emam, Doaa A. Semida, Ensaf A. El-Full, Bothaina Y. Mahmoud, Ali M. Abdel-Azim, Shaaban Saad Elnesr

**Affiliations:** Department of Poultry Production, Faculty of Agriculture, Fayoum University, Fayoum, 63514 Egypt

**Keywords:** Sex ratio, Body weight, Selection, Dam, Japanese quail

## Abstract

Offspring sex ratios in avian species are of significant scientific interest, with implications for evolutionary biology and poultry production. This study investigated sex ratios in Japanese quail (*Coturnix japonica*), a valuable model for other poultry species due to its rapid generation interval. The study examined the impact of selection over generations, age at first egg (AFE), and body weight at AFE (BW_AFE_) on offspring sex ratios. The dataset included 4,282 Japanese quail records from 968 dams over eight generations, comprising two lines: one selected for high growth rate during 1–21 days of age and an unselected control line. Offspring sex ratio data were categorized based on dam characteristics: AFE (early: <48 days, medium: 48–52 days, late: >52 days) and BW_AFE_ (low: <249 g, medium: 249–268 g, heavy: >268 g). These categories represent below average, average, and above average values for each parameter, respectively. Analyses were done on pedigree and hatching records from two lines of selected and control quails. The chi square and logistic regression analyses exhibited insignificant associations between the examined predictor variables (generation, line, AFE, and BW_AFE_) and the sex ratio outcome in Japanese quail. Therefore, it can be concluded that the proportion of male and female offspring quail in the flock is statistically equal. However, regarding the BW_AFE_ categories the residual analyses revealed a potential tendency toward a male-biased sex ratio within the medium category also, they suggest potential tendencies toward male-biased (eighth generation) and female-biased (sixth generation) sex ratios that warrant further investigation.

## Introduction

The sex ratio of offspring in avian populations plays a crucial role in understanding population dynamics and has significant implications for both scientific research and practical poultry farming (Prior et al. 2011). In this context, Japanese quail (*Coturnix coturnix japonica*) serves as an excellent model species for studying sex allocation due to its unique life history characteristics, including small body size, short generation interval, quick sexual maturity, fast growth rate, and significant egg production (Özsoy and Aktan 2011; Mahmoud et al. 2015; Batool et al. 2023). These features make Japanese quail an ideal candidate for various investigations on productive indices (Abou Khadiga et al. 2016; Emam et al. 2023).

There are two theories suggesting how the male and female distribution ratios are in the offspring. The first is Fisher’s theory, which stated that the natural selection should favor investments in offspring with equal sex ratio: any deviation from equality should not be nominated due to the negative frequency-dependent selection (the simple deviation from equality cancels out in the population, thus the best approach is to create offspring with an equal ratio between the sexes) (Fisher 1930). The second is Trivers-Willard hypothesis, which demonstrated that the selection under various maternal conditions should encourage deviations from the equal sex ratio of offspring. This is due to the fact that males are more severely affected by maternal conditions than females are, males benefit more from good conditions but suffer more in poor conditions, while females have more stable reproductive success across varying conditions (Trivers and Willard 1973). Birds have a special ability to influence primary sex ratios because females are the heterogametic sex (carrying both sex chromosomes, W and Z), and therefore have the ability to directly control the sex of offspring and possibly modify the primary sex ratio (Sheldon 1998). Aslam (2014) clarified that a better understanding of mechanisms underlying sex ratio bias may have significant effects for poultry farming, as optimizing sex ratios in commercial production could lead to more efficient resource allocation and improved economic outcomes.

For instance, in laying hen operations, a higher proportion of female chicks is desirable to avoid the ethical and economic challenges associated with culling male day-old chicks (Krautwald-Junghanns et al. 2018). Conversely, in meat production, a balanced or slightly male-biased ratio might be preferred due to the faster growth rates of males (Laughlin 2007). Several studies across various avian species have focused on dam-related factors and provided for primary sex ratio adjustment (Komdeur and Pen 2002). While some factors influencing offspring sex ratio are subject to human intervention, such as diet (Rosenfeld and Roberts 2004) and external hormonal manipulation (Groothuis et al. 2005), naturally occurring factors like dam age and weight at sexual maturity remain less understood (Alonso-Alvarez and Velando 2003; Aslam et al. 2013). Because of numerous confounding elements in correlative studies such as species effects of parental age and environmental change as in which sex is chromosomally determined, it is challenging to pinpoint the exact conditions that are predictably related to biased sex ratio (Nager et al. 1999).

This knowledge gap presents a significant challenge in sex ratio investigations and highlights the need for further research in this area. By focusing on these naturally occurring factors, we seek to generate valuable information on the mechanisms underlying sex ratio bias in this species. Therefore, the present study aims to address this research gap by investigating the effects of specific independent categorical variables (generation, line comprising two lines: one selected for high growth rate during 1–21 days of age and an unselected control line, age at first egg (AFE), and body weight at first egg (BW_AFE_)) on the sex ratio of Japanese quail offspring.

## Materials and methods

### Experimental design and animal dataset

The study protocol was agreed by the Fayoum University Institutional Animal Care and Use Committee (FU-IACUC), Egypt (approval no. AEC2324). The experimental design and analysis were intended to study the effects of selection, successive generations, age and body weight at maturation on offspring sex ratios. The dataset consisted of 4,282 Japanese quail records (3,093 in the selected line (SL) and 1,189 in the control line (CL)) obtained from 968 dams across eight generations, which were reared in the Poultry Research Center at the Faculty of Agriculture, Fayoum University in Egypt. The analyses were conducted on pedigree and hatching records across the eight generations, obtained from two lines of quails (SL and CL). The setting and hatching records of each of the 968 dams which had full record of no mortality of their offspring (734 selected dams and 234 control dams) were analyzed in order to avoid any effects of mortality on offspring’ sex ratio. The study focused on variables including successive generations (across eight generations), selection (SL *vs* CL), age at maturation, body weight at maturation, and offspring sex ratios. The analysis aimed to investigate how these variables influenced offspring sex ratios in Japanese quail.

Aggregated breeding values for a selection criterion were estimated in two lines of Japanese quail as a by-product of calculating heritability estimates using restricted maximum likelihood (REML) procedures via the WOMBAT program software (Meyer 2007). The selection criterion was high growth rate during the first 21 days of age. Two lines were maintained:

1. Selected line (SL): This line underwent selection for eight successive generations based on the estimated aggregated breeding values for the selection criterion.
2. Control line (CL): this line was kept under random mating without any selection pressure applied.

### Quail husbandry and management

The selected breeders of quail (*Coturnix coturnix japonica*) were housed in breeding cages. For mating purposes, two females were randomly assigned to each male. Mating of close relatives was avoided to decrease the rate of inbreeding depression. A pedigree system was implemented by daily collecting eggs from each quail family when their females were 11–14 weeks old, based on the shell color and patterns of each female. The eggs were incubated once a week under the artificial incubation conditions. The hatchlings chicks were wing banded using little plastic bands, and brooded on the floor with wheat chaff litter until 35 days of age. Quails were provided with ad libitum diet containing of 2900 kcal of metabolizable energy and 24% crude protein from hatching day up to 35 days of age, as recommended by NRC (1994). From 35 days of age until the end of the egg-laying period, quails breeders were given a breeder diet containing 20% crude protein, 2,900 kcal of metabolizable energy, 2.25% calcium, and 0.43% available phosphorus .

### Sexing Japanese quail chicks

**Fig. 1:**
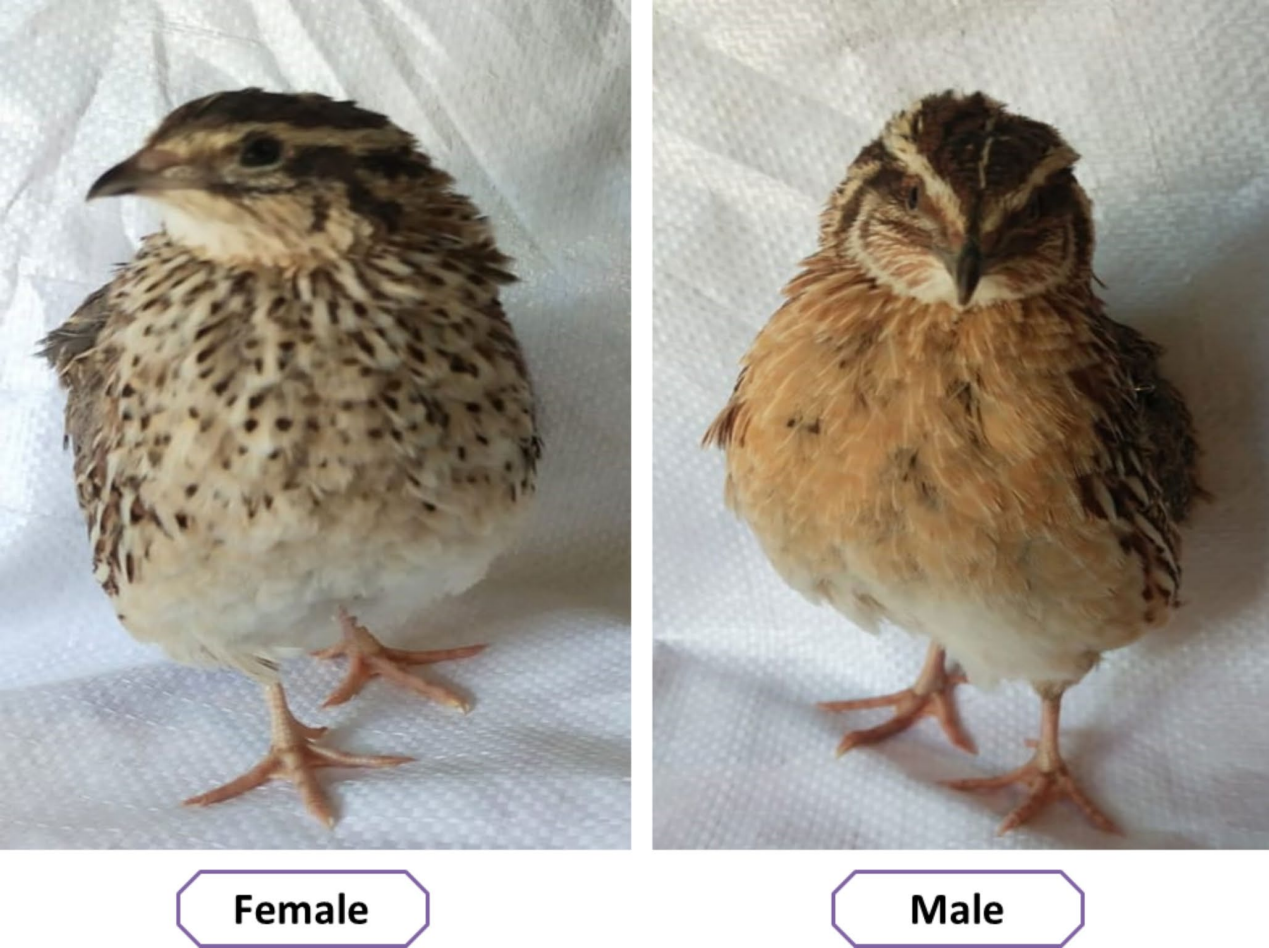
Quail sex determination according to breast plumage color

Quail chicks were sexed as early as 21 days of age by examining their feather color, which is distinctive for males and females. Females have a plumage color on the throat and breast that is cinnamon, while males have a rusty brown color (Fig. 1). At 35 days of age, all birds were transferred to individual laying cages and their sex was confirmed. This was done to ensure accurate pedigree identification. The birds were kept under consistent hygienic and environmental conditions throughout the study.

### Statistical analyses

Descriptive statistics for age and body weight of the dams at first egg (AFE in days and BW_AFE_ in grams) that may have affected sex ratio were calculated for overall means, for the SL and CL in Japanese quail are shown in Table 1. Offspring sex ratio data were categorized based on dam characteristics: AFE (early: <48 days, medium: 48–52 days, late: >52 days) and BW_AFE_ (low: <249 g, medium: 249–268 g, heavy: >268 g). These categories represent below average, average, and above average values for each parameter, respectively.

**Table 1:** Descriptive statistics for the dams-related variables that may affect offspring sex ratio in Japanese quail

Trait	Classes	Numbers of dams	Mean	Minimum	Maximum	Standard error
AFE, days	Early	347	43.00	33.00	47.00	0.12
	Medium	295	50.00	48.00	52.00	0.05
	Late	326	58.00	53.00	84.00	0.18
	Total	968				
BW_AFE_, g	Low	307	233.00	183.00	248.00	0.44
	Medium	341	259.00	249.00	268.00	0.20
	Heavy	320	284.00	268.00	352.00	0.44
	Total	968				

SAFE = age at first egg (days); BW_AFE_ = body weight at first egg (g)

Chi-Square and logistic regression analyses were performed to investigate the effects of the independent categorical variables (generation, line, AFE and BW_AFE_) on the sex ratio as an outcome variable using SPSS (2008) statistical software. The logistic regression model is based on the logit transformation of the probability of the outcome variable.

$${\rm{logit}}\left( {\rm{p}} \right)\,{\rm{ = }}\,{{\rm{\beta }}_{\rm{0}}}\,{\rm{ + }}\,{{\rm{\beta }}_{\rm{1}}}{{\rm{X}}_{\rm{1}}}\,{\rm{ + }}\,{{\rm{\beta }}_{\rm{2}}}{{\rm{X}}_{\rm{2}}}\,{\rm{ + }}\,{{\rm{\beta }}_{\rm{3}}}{{\rm{X}}_{\rm{3}}}\,{\rm{ + }}\,{{\rm{\beta }}_{\rm{4}}}{{\rm{X}}_{\rm{4}}}\,{\rm{ + }}\,{\rm{\varepsilon }}$$

Where: p = probability of the outcome, β₀ = intercept (constant), β₁, β₂, β₃, β₄ = regression coefficients, X₁ = Generation (G1 to G8), X₂ = Line (CL or SL), X₃ = Age at first egg (AFE) category, X₄ = Body weight at first egg (BW_AFE_) category, and ε = error term.

For Chi-Square statistics, adjusted and standardized residuals for each cell were calculated and discussed to evidence which discrepancies between observed and expected values are larger than those might be expected by chance. The significance level for all statistical tests was set at *p* = 0.05.

## Results

### Effect of generation of selection on sex ratio of Japanese quail

As presented in Table 2, the chi-square test was used to analyze the sex ratio data across different selection generations. The omnibus chi-square statistic was 3.919 with a corresponding p-value of 0.789, indicating that selection generations did not significantly affect the sex ratio distribution than the expected 1:1 sex ratio. To further investigate potential deviations within specific generations, residuals were examined. Generations 6 and 8 exhibited the largest standardized and adjusted residuals. For generation 6, these values were 0.536 and 0.541, while for generation 8, they were 0.981 and 0.972, respectively. The corresponding Pearson residuals were 0.792 and 1.460. These residuals indicate the degree of deviation between observed and expected frequencies in our chi-square analysis. Standardized and adjusted residuals are expressed in standard deviation units, with values exceeding ± 1.96 typically considered significant at α = 0.05. Although our observed values do not surpass this threshold, they are notably larger than those for other generations, particularly for generation 8. This suggests potential tendencies toward female-biased (sixth generation) and male-biased (eighth generation) sex ratios. While not statistically significant at the conventional 0.05 level, these larger residuals indicate greater deviations from expected values compared to other generations, warranting further investigation in future studies.

**Table 2:** Chi-square, standardized and adjusted residuals of sex ratio data according to selection generations

**Offspring**		G	Dam numbers		Sex			G	Dam numbers	Sex	
					**Female**	**Male**				Female	Male
Observed		G_1_	163		361	361		G_5_	96	284.0	304.0
Expected					358.10	363.90				291.70	296.30
% within					50.00	50.00				48.30	51.70
Standardized residual					0.151	-0.150				-0.445	0.445
Cell chi-square					0.022	0.022				0.198	0.198
Adjusted residual					0.233	-0.233				-0.681	0.681
Observed		G_2_	175		374	362		G_6_	86	162	151
Expected					365.1	370.90				153.3	157.7
% within					50.80	49.20				51.80	48.20
Standardized residual					0.466	-0.463				0.541	-0.536
Cell chi-square					0.217	0.214				0.292	0.287
Adjusted residual					0.722	-0.722				0.792	-0.792
Observed		G_3_	108		234	245		G_7_	126	261	250
Expected					237.6	241.4				253.50	257.50
% within					48.90	51.10				51.10	48.90
Standardized residual					-0.233	0.231				0.473	-0.469
Cell chi-square					0.054	0.053				0.223	0.219
Adjusted residual					-0.348	0.348				0.709	-0.709
Observed		G_4_	123		238	242		G_8_	91	210.00	243.0
Expected					238.10	241.90				224.70	228.30
% within					49.60	50.40				46.40	53.60
Standardized residual					-0.006	0.006				-0.981	0.972
Cell chi-square					0.0003	0.0003				0.962	0.944
Adjusted residual					-0.009	0.009				-1.460	1.460
Observed		Total		968	2,124	2,158					
Expected					2,124	2,158					
% within					49.60	50.40					
χ^2^		3.919									
Likelihood Ratio		3.922									
***Probability***		***0.789***									

G = generation

### Effect of line on sex ratio of Japanese quail

Data shown in Table 3 investigated potential differences in sex ratio between the CL and SL lines by performing a chi-square analysis. The omnibus chi-square statistic was 0.067, with a corresponding p-value of 0.796. This result indicates no significant deviation from the expected 1:1 sex ratio when comparing the two lines. Examination of the standardized residuals revealed relatively small values for both the CL (-0.154) and SL (0.096) lines. Furthermore, the adjusted residuals were (-0.224-0.257) for the CL line and (-0.257-0.257) for the SL line indicating that the genetic factors associated with these lines did not significantly skew the mechanisms underlying sex ratio determination.

**Table 3:** Chi-square, standardized and adjusted residuals of sex ratio data according to selected and control lines

Line	Dam numbers	Offspring	Sex	
			Female	Male
CL	234	Observed	586	603
		Expected	589.80	599.20
		% within	49.30	50.70
		Standardized residual	-0.154	0.154
		Cell chi-square	0.023	0.023
		Adjusted residual	-0.224	0.257
SL	734	Observed	1538	1555
		Expected	1,534.20	1,558.8
		% within	49.70	50.30
		Standardized residual	0.096	-0.095
		Cell chi-square	0.009	0.009
		Adjusted residual	0.257	-0.257
Total	968	Observed	2,124	2,158
		Expected	2,124	2,158
		% within	49.60	50.40
χ^2^		**Value**	***Probability***	
		0.067	***0.796***	
Continuity Correction*		0.050	***0.823***	
Likelihood Ratio		0.067	***0.796***	
Fisher’s Exact Test			***0.811***	

CL = control line and SL = selected line

* Continuity Correction refers to Yates’ correction for continuity, applied to improve the accuracy of the chi-square approximation for 2 × 2 contingency tables

### Effect of dam’s age and body weight at first egg on sex ratio of Japanese quail

The chi-square analyses were conducted to investigate potential differences in sex ratio across different categories of AFE and BW_AFE_ as shown in Tables 4 and 5. For the AFE categories (Early, Medium, and Late), the omnibus chi-square statistic was 0.055, with a corresponding p-value of 0.973. Examination of the standardized and adjusted residuals revealed minimal values for all AFE categories, indicating no significant deviations from the expected sex ratios within each category. Regarding the BW_AFE_ categories (Low, Medium, and Heavy), the omnibus chi-square statistic was 3.834, with a p-value of 0.147. However, the residual analysis revealed a potential tendency toward a male-biased sex ratio within the Medium category. The standardized residual for the Medium category was − 1.880, and the adjusted residual was − 1.894, suggesting a deviation from the expected sex ratio that may need further investigation to clarify the underlying mechanisms influencing sex ratio determination in Japanese quail populations. Chi square analyses for different categories of AFE and BW_AFE_ indicated that these categories did not significantly affect the sex ratio distribution compared with the expected 1:1 sex ratio.

**Table 4:** Chi-square, standardized and adjusted residuals of sex ratio data for age at first egg (AFE) categories (early, medium and late)

AFE categories	Dam numbers	Offspring	Sex	
			Female	Male
Early	325	Observed	755	760.0
		Expected	751.50	763.50
		% within	49.80	50.20
		Standardized residual	0.128	-0.127
		Cell chi-square	0.016	0.016
		Adjusted residual	0.224	-0.224
Medium	312	Observed	648	660
		Expected	648.80	659.20
		% within	49.50	50.50
		Standardized residual	-0.031	0.031
		Cell chi-square	0.0009	0.0009
		Adjusted residual	-0.053	0.053
Late	331	Observed	721	738
		Expected	723.7	735.3
		% within	49.40	50.60
		Standardized residual	-0.099	0.099
		Cell chi-square	0.009	0.009
		Adjusted residual	-0.174	0.174
Total	968	Observed	2,124	2,158
		Expected	2,124	2,158
		% within	49.60	50.40
χ^2^		0.055		
**Likelihood Ratio**		0.055		
**Probability**		***0.973***		

**Table 5:** Chi-square, standardized and adjusted residuals of sex ratio data for body weight at first egg (BW_AFE_) categories (low, medium and heavy)

BW_AFE_ categories	Dam numbers	Offspring	Sex	
			Female	Male
Low	315	Observed	725	722
		Expected	717.80	729.2
		% within	50.10	49.90
		Standardized residual	0.268	-0.268
		Cell chi-square	0.071	0.071
		Adjusted residual	0.468	-0.468
Medium	324	Observed	670	739
		Expected	698.9	710.1
		% within	47.60	52.40
		Standardized residual	-1.880	1.880
		Cell chi-square	3.53	3.53
		Adjusted residual	-1.894	1.894
Heavy	329	Observed	729	697
		Expected	707.30	718.7
		% within	51.10	48.90
		Standardized residual	0.808	-0.808
		Cell chi-square	0.652	0.652
		Adjusted residual	1.516	-1.404
Total	968	Observed	2,124	2,158
		Expected	2,124	2,158
		% within	49.60	50.40
χ^2^		3.834		
Likelihood Ratio		3.835		
Probability		***0.147***		

### Logistic regression of statistical model in sex ratio of Japanese quail on the basis of generation, line, age at first egg (AFE) and body weight at first egg (BWAFE)

As shown in Table 6, the logistic regression analysis did not reveal any significant associations between the examined predictor variables (generation, line, AFE and BW_AFE_) and the sex ratio outcome. The p-values for all predictor variables were well above the conventional significance level of 0.05 as p-value ranging from 0.335 to 0.974 for generation, 0.202–0.953 for lines, 0.128–0.960 for age at first egg categories, and 0.429–0.836 for body weight at first egg categories. Additionally, the Exp(B) values for the predictor categories ranged from 0.866 to 1.186. This finding suggests that, within the scope of the current study, these variables did not significantly influence the sex ratio of the offspring. Briefly, in the current Japanese quail’s population, statistical analyses for independent categorical variables (generation, line, AFE and BW_AFE_) indicated that these variables did not significantly affect the sex ratio distribution than the expected 1:1 sex ratio.

**Table 6:** Logistic regression of statistical models to explain variation in sex ratio of Japanese quail on the basis of generation, line, age at first egg (AFE) and body weight at first egg (BW_AFE_)

Item	B	SE	Wald	Sig.	Exp. (B)
**Generation**			3.143	0.871	
G_1_	0.040	0.198	0.040	0.842	1.040
G_2_	0.112	0.178	0.398	0.528	1.119
G_3_	-0.006	0.192	0.001	0.974	0.994
G_4_	0.044	0.179	0.060	0.806	1.045
G_5_	0.006	0.182	0.001	0.973	1.006
G_6_	0.186	0.210	0.785	0.376	1.204
G_7_	0.165	0.172	0.913	0.339	1.179
G_8_	0.155	0.181	0.654	0.335	1.167
**Line**			1.759	0.415	
CL	0.007	0.125	0.003	0.953	1.007
SL	-0.112	0.088	1.631	0.202	0.894
**AFE**			3.207	0.361	
Early	0.170	0.403	0.178	0.673	1.186
Late	-0.006	0.113	0.003	0.960	0.994
Medium	0.144	0.095	2.317	0.128	1.155
**BW**_**AFE**_			0.886	0.829	
Heavy	-0.143	0.405	0.125	0.723	0.866
Low	0.020	0.098	0.043	0.836	1.021
Medium	0.089	0.112	0.626	0.429	1.093
Constant	-0.131	0.194	0.452	0.501	0.877

CL = control line, SL = selected line, B = regression coefficient, SE = standard error, Sig. = significance and Exp. = Euler’s number (~ 2.71828….)

## Discussion

In most animal species, sex is one of the primary axes of behavioral and morphological difference within a population (Radder et al. 2007). Birds are interesting themes because the female is the heterogeneous sex, which means that they are likely to determine the ratio of sex in their offspring. In a number of species, sex ratio biases with respect to maternal or environmental impacts have been verified (Goerlich-Jansson et al. 2013). Theoretically, producers can guess a 1:1 ratio of female to male offspring in a population or breeding herd. Hays (1945) was the first to investigate the primary sex ratio of Rhode Island Red chickens of 49.7 among 870 chicks from 39 perfect record females but the physiological mechanisms driving biases in primary avian sex ratio are still unknown. Landauer (1957) offered evidence on Black Minorca and White Leghorn progenies, clearly demonstrating that the primary sex ratio was equal, whereas, Coles (1956) had earlier established a significant departure from the equality of primary sex ratio in Leghorn stocks. Feng et al. (2006) clarified that the sex ratio of the entire flock that was analyzed in their study had only a little excursion, thus accorded with the expected sex ratio. Therefore, several studies have provided divergent sex ratio data.

The chi-square and logistic regression analyses conducted in this study aimed to investigate potential deviations from the expected 1:1 sex ratio across various variables, including selection generations, lines (CL and SL), AFE categories, and BW_AFE_ categories. The overall chi-square tests did not detect significant deviations from the expected 1:1 sex ratio when considering all selection generations collectively, comparing the CL and SL lines, or across different AFE categories. Similarly, no significant differences in sex ratio were observed across the different AFE categories. These findings suggest that, within the scope of the present study, the sex ratio remained balanced across multiple selection cycles, between the two lines, and across different ages at which females initiated egg production. However, the residual analyses revealed potential tendencies toward sex ratio biases in specific contexts. For instance, the sixth and eighth generations exhibited relatively large standardized and adjusted residuals, suggesting potential female-biased and male-biased sex ratios, respectively. These findings warrant further investigation and indicate that females may have some capacity to influence offspring sex ratios naturally. Maternal skewing of offspring sex ratio could have vital agricultural implications, since chicks of one gender may be desired over the other (Rosenfeld and Roberts 2004). For instance, in laying hen, female chicks is desirable to avoid the ethical and economic challenges associated with culling male day-old chicks (Krautwald-Junghanns et al. 2018), whereas, in meat production, a balanced or slightly male-biased ratio might be preferred due to the faster growth rates of males (Laughlin 2007).

The current study contributes to the ongoing dialogue about sex ratio allocation in birds, specifically in Japanese quail. While we did not find significant overall deviations from the expected 1:1 ratio, the subtle biases observed in certain contexts highlight the complexity of this phenomenon. These findings are consistent with those of Champion (1960) who revealed that the primary sex ratio did not vary significantly from equality in the tested populations. Additionally, Cholewa et al. (2021) clarified that the female-to-male ratio was 195 (48.9%): 204 (51.1%), a result not statistically different from 50:50 ratio (*χ*^2^ = 0.206).

The physiological mechanisms driving biases in primary avian sex ratio remain largely unknown. Some researchers propose that involvement of meiotic drive has been suggested as a potential factor in sex bias, as interference with meiosis has been shown to affect sex ratios (Aslam 2014). Environmental conditions and parental traits have been implicated in offspring sex ratio variations across bird species (Cholewa et al. 2021). Factors such as maternal body condition, age, and social environment have been explored as potential influences on sex allocation. In the current study, the dam body weight at first egg did not change the offspring’s sex. However, within the BW_AFE_ categories, the Medium category displayed larger residual values, indicating a potential tendency toward a male-biased sex ratio. The results of Nager et al. (1999) provided experimental evidence of adaptive and facultative adjustments of sex ratio in response to alterations in maternal conditions in wild avian populations. In the current study, we extend the experimental evaluation of natural sex ratio allocation to check whether dams change sex ratio of offspring. Skewing sex ratio of offspring can take place when any factor predictably but differently impacts the number of males vs. females. One such factor that connects with the sex ratio is the parents’ body condition (Alonso-Alvarez 2006). Erikstad et al. (2009) illustrated that parents have control over their young’s sex and can increase the ratio of the sex that is most beneficial to their fitness. Alterations in body condition reflect negative energy balance and should therefore be considered as a main factor influencing the dam physiology.

The beginning of sexual maturation is a feature that is extremely significant in terms of both evolution and economics. A number of morphological traits were shown to be significantly predicted by the start of sexual maturity (Wright et al. 2012). The current results displayed that the offspring sex ratio is not affected by the age of dam at first egg. Langen et al. (2018) found no influences of the maternal social environment on the sex ratio of offspring in Japanese quails, therefore does not necessarily adjust offspring physiology and sex allocation, even if it leads variances in maternal physiology. It is believed that gender-specific maternal effects originated as adaptations to increase the fitness of female and male offspring for their expected environment (Langen et al. 2018). Paternal age has not been found to significantly influence offspring sex ratios across species (Booksmythe et al. 2017). In Blackbird population, Cholewa et al. (2021) discovered that the general sex ratio did not statistically deviate from 50:50, but they also noted that the parental age may have a greater contribution to reproductive success and shaping offspring. However, although the age at sexual maturity affects reproduction (Tan et al. 2021), it did not affect the sex ratio in the results of the current study.

Japanese quail are developed by estimating different genetic and phenotypic parameters, and this allows specific lines to be created through selection programs (Mahmoud et al. 2015, 2023; Farahat et al. 2018), which is accompanied by improvements in body weights at different ages, and egg production traits (Abou Khadiga et al. 2018; Semida et al. 2019). Knowledge of the sex ratio of offspring is necessary for understanding how individual characteristics alter from one generation to another in response to selection. If there is no relationship between this ratio under selection generations, the overall response to our concept will confirm beyond any doubt that selection does not affect this cosmic proportion of preserving offspring naturally. This will further our understanding of the evolutionary consequences of the sex ratio of hatchlings in the different flocks.

The current analyses of dams, hatching records and sex ratio data for different generations were undertaken to deliver further information on sides of this problem (emphasizing the gender rate for offspring in Japanese quail). The current work showed that although the background of the dams is different and the upbringing is different, it did not affect the sexual ratio of the offspring. The overall female-to-male ratio in these populations of Japanese quail was not statistically different from 50:50 ratio, and this was confirmed by more than one factor and using a large number of dams (*n* = 968), which increases the accuracy of the information. Therefore, it could be sure that the relationship between sex ratio of offspring was equal (50:50) although different factors related to parents.

### Limitations and future research directions

As we continue to unravel the mysteries of sex allocation in birds, we acknowledge certain limitations. The study’s duration of 8 generations, while substantial, may not capture longer-term trends. Our focus on specific variables and a particular quail line may limit generalizability. Despite these constraints, we believe our findings provide a robust foundation for understanding sex ratio dynamics in quail breeding populations under current management practices. These limitations offer valuable directions for future research. Future research should explore additional environmental factors like temperature, photoperiod, and dietary composition. Molecular studies investigating sex determination mechanisms and potential meiotic drive in avian species would further advance our understanding of this intricate process.

## Conclusion

Investigating and potentially influencing offspring sex ratios is crucial in poultry farming, with significant implications for resource allocation and economic outcomes. In laying hen operations, for example, a higher proportion of female chicks is preferable to mitigate the ethical and economic challenges of culling male day-old chicks. Although, the present study did not find significant associations between the examined variables and sex ratio, continued monitoring and exploration of other factors are recommended to validate these findings and identify potential drivers of sex ratio biases, if any, in future studies or under different environmental or experimental conditions.

## Data Availability

The data that support the findings of this study are available on request from the corresponding author.

## Declarations

### Animal welfare statement

The study protocol was agreed by the Fayoum University Institutional Animal Care and Use Committee (FU-IACUC), Egypt (approval no. AEC2324).
